# Characterization of Arginine Catabolism by Lactic Acid Bacteria Isolated from Kimchi

**DOI:** 10.3390/molecules23113049

**Published:** 2018-11-21

**Authors:** Hyelyeon Hwang, Jong-Hee Lee

**Affiliations:** Research and Development Division, Advanced Process Technology and Fermentation Research Group, World Institute of Kimchi, Gwangju 61755, Korea; hyelyeon@wikim.re.kr

**Keywords:** arginine, citrulline, ornithine catabolism, fermentation, flavor, kimchi, lactic acid bacteria, *Lactobacillus brevis*, *Weissella confusa*

## Abstract

Kimchi fermentation depends on diverse lactic acid bacteria, which convert raw materials into numerous metabolites that contribute to the taste of food. Amino acids and saccharides are important primary metabolites. Arginine is nearly exhausted during kimchi fermentation, whereas the concentrations of other amino acids are reported not to increase or decrease dramatically. These phenomena could imply that arginine is an important nutritional component among the amino acids during kimchi fermentation. In this study, we investigated the arginine-catabolism pathway of seven lactic acid bacteria isolated from kimchi and evaluated the products of arginine catabolism (citrulline and ornithine) associated with the bacteria. The arginine content dramatically decreased in cultures of *Lactobacillus brevis* and *Weissella confusa* from 300 μg/mL of arginine to 0.14 ± 0.19 and 1.3 ± 0.01 μg/mL, respectively, after 6 h of cultivation. Citrulline and ornithine production by *L. brevis* and *W. confusa* showed a pattern that was consistent with arginine catabolism. Interestingly, *Pediococcus pentosaceus*, *Lactobacillus plantarum*, *Leuconostoc mesenteroides*, and *Leuconostoc lactis* did not show increased citrulline levels after arginine was added. The ornithine contents were higher in all bacteria except for *L. lactis* after adding arginine to the culture. These results were consistent with the absence of the arginine deiminase gene among the lactic acid bacteria. Arginine consumption and ornithine production were monitored and compared with lactic acid bacteria by metagenomics analysis, which showed that the increment of ornithine production correlated positively with lactic acid bacteria growth.

## 1. Introduction

Kimchi is a representative fermented vegetable food. It composed of diverse ingredients, such as cabbage, garlic, ginger, and pepper. It is known that kimchi consumption can be beneficial for health [1,2]. Kimchi contains diverse microflora that produce nutritious metabolites [1]. It comprises various vegetables, lactic acid bacteria, and metabolites produced by the fermentation of ingredients and the growth of lactic acid bacteria. Source tracking of kimchi has shown that its ingredients of kimchi affect the ontogeny of lactic acid bacteria. For example, garlic is an important source of lactic acid bacteria in kimchi [3,4,5,6], and red pepper has been reported as a source of *Weissella* sp. [7].

Kimchi contains diverse metabolites produced by bacteria, as demonstrated by an analysis of the bacterial population during fermentation and of adding bacteria to kimchi [4,8,9]. Amino acids (aas) are essential for the survival of living organisms. The aa contents in kimchi have been characterized previously [10,11]. The aa contents were found to change during fermentation and differed depending upon the raw ingredient composition in kimchi. Glutamic acid and aspartic acid are the most abundant aas in kimchi. It has been reported that overall aa content slightly increased during fermentation. The total aa content in kimchi slightly increased due to the degradation of proteinaceous components such as jeotgal (fish sauce) during fermentation [12]. Interestingly, among the aas, arginine content constantly decreased in kimchi over time during fermentation.

Those findings implied that arginine is an important nutritional source for bacteria during kimchi fermentation. Indeed, arginine also serves as an important energy resource in the bacteria by participating in the arginine deiminase (ADI) pathway. Arginine catabolism is beneficial for bacteria in that it improves bacterial growth and regulates pH during fermentation [13,14,15].

The ADI pathway is composed of ADI, ornithine transcarbamylase (OTC), and carbamate kinase (CK). The ADI pathway catalyzes the conversion of arginine to citrulline, ammonium, and carbon dioxide, while generating ATP from ADP and phosphate [16]. Citrulline can be further metabolized into ornithine and carbamoyl-phosphate [14].

Ammonia is strongly basic and acts as a signaling molecule with yeast and ammonia-oxidizing bacteria [17,18]. Thus, arginine degradation could influence the growth and adaptation of bacteria under acidic conditions and further influence the emergence of other bacterial groups during kimchi fermentation.

Bacterial communities change during fermentation, which may reflect the production of organic acids that lower pH [3,4,5]. Metabolite formation in kimchi mainly depends on bacterial composition and the associated fermentation steps and metabolic pathways carried out by each bacterial species.

The high catabolism of arginine during kimchi fermentation suggests the importance of arginine in kimchi fermentation and that it may influence bacterial growth during kimchi fermentation. However, little is known regarding arginine catabolism in kimchi by kimchi-originated bacteria, including citrulline and ornithine production. Thus, it is important to characterize the arginine-catabolism pathway of kimchi-originated lactic acid bacteria to evaluate arginine consumption associated with kimchi fermentation.

In this study, we investigated the arginine-catabolism pathway of lactic acid bacteria isolated from kimchi to evaluate arginine consumption in kimchi and its relationship with lactic acid bacteria in the fermentation environment.

## 2. Results

### 2.1. Arginine Catabolic Activity of Lactic Acid Bacteria

To validate the utilization of arginine by lactic acid bacteria, the bacteria were cultivated and the arginine content was correlated with the growth of bacteria. The lactic acid bacteria *Lactobacillus brevis*, *Pediococcus pentosaceus*, *Lactobacillus plantarum*, *Leuconostoc lactis*, *Lactobacillus sakei*, *Leuconostoc mesenteroides*, and *Weissella confusa* were previously isolated from homemade kimchi and characterized in terms of 2-hydroxyisocaproic acid (HICA) production in leucine catabolism [5]. The bacteria were inoculated into De Man, Rogosa, and Sharpe (MRS) medium, and the arginine contents were measured during bacterial growth.

**Figure 1:**
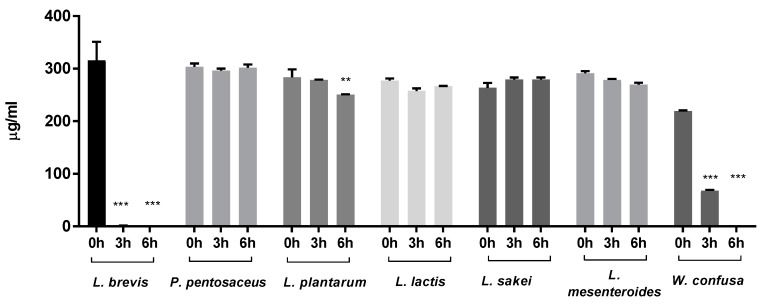
Arginine consumption by lactic acid bacteria isolated from kimchi. Bacteria were cultivated in De Man, Rogosa, and Sharpe (MRS) medium, and the arginine contents in the media over time were quantified by liquid chromatography-mass spectrometry (LC-MS, ** *p* < 0.01, *** *p* < 0.001). *L. brevis*: *Lactobacillus brevis*; *P. pentosaceus: Pediococcus pentosaceus*; *L. plantarum: Lactobacillus plantarum*; *L. lactis*: *Leuconostoc lactis*; *L. sakei*: *Lactobacillus sakei*; *L. mesenteroides*: *Leuconostoc mesenteroides*; *W. confusa*: *Weissella confusa*.

The MRS medium samples initially contained 200–300 μg/mL (approximately 1.1–1.7 mM) of arginine. This concentration decreased to 67 ± 1.8 μg/mL after 3 h and was almost exhausted after 6 h of cultivation with *W. confusa*. *L. brevis* cultures had arginine concentrations of 0.60 ± 0.85 μg/mL and 0.14 ± 0.19 μg/mL after 3 h and 6 h of fermentation, respectively. The arginine concentration of the *L. plantarum* and *L. mesenteroides* cultures decreased to 250.5 ± 0.7 μg/mL and 269.5 ± 3.5, respectively (Figure 1).

### 2.2. The ADI System of Lactic Acid Bacteria

The distribution of ADI, OTC, and CK was investigated. The aa and gene sequences of ADI and OTC were obtained from the UniProt Protein Database and compared using the CLC genomics workbench program, version 7.5. The distributions of ADI and OTC in lactic acid bacteria are listed in Table 1 and Table 2.

**Figure 2:**
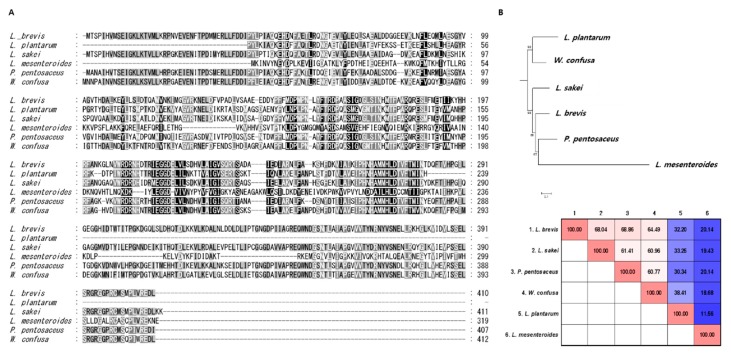

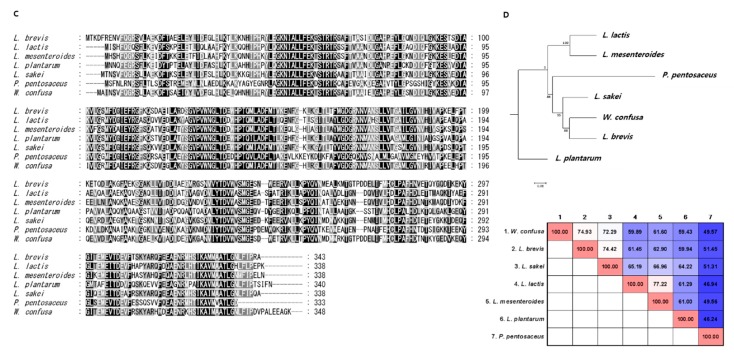
Comparison of ADI and OTC sequences between different lactic acid bacteria: (**A**) alignments of ADI amino acid (aa) sequences; (**B**) phylogenic tree of ADI; (**C**) alignments of OTC aa sequences; (**D**) phylogenic tree of OTC. The homology of each sequence is represented in a heatmap. Detailed information for the protein sequences (including accession numbers) is provided in Table 1.

Comparison of ADI sequences revealed a region containing highly conserved aa sequences (aas 220–290). The overall aa sequence similarity of ADI ranged from 20.1% to 68.8% among the different bacterial species (Figure 2A,B). Comparison of the OTC sequences showed that aas 130–177 were highly conserved and shared 49.5%–77.2% aa sequence similarity among the different species studied (Figure 2C,D). The ADI and CK proteins were not annotated for *L. lactis*, and CK was not annotated for *L. mesenteroides*.

### 2.3. Arginine, Citrulline, and Ornithine Production by Lactic Acid Bacteria

**Figure 3:**
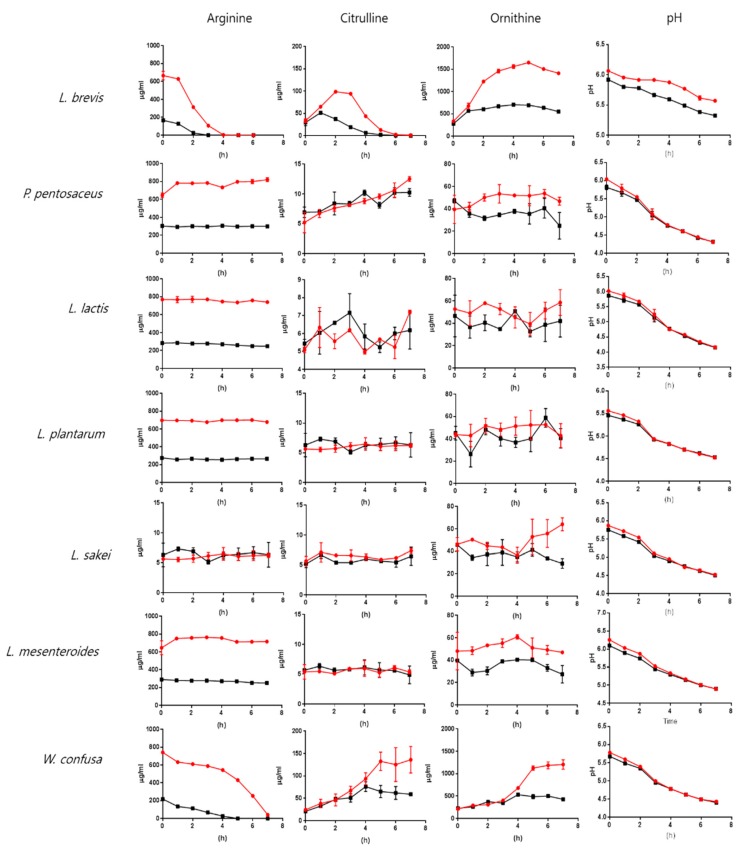
Analysis of citrulline and ornithine production by lactic acid bacteria. MRS medium samples were prepared by adding 500 μg/mL arginine (the red line), and the bacteria were cultivated at 30 °C. The arginine, citrulline, and ornithine contents were measured by LC-MS. *L. brevis* and *W. confusa* showed the highest arginine-catabolism activity and the highest production of citrulline and ornithine.

Arginine-dependent production of citrulline and ornithine was investigated by cultivating lactic acid bacteria in MRS medium or MRS medium containing extra arginine. The metabolites were quantified by liquid chromatography-mass spectrometry (LC-MS) in multiple reaction-monitoring (MRM) mode. Arginine (500 μg/mL) was added to the MRS medium, and the citrulline and ornithine concentrations were compared to those in the MRS medium without added arginine (Figure 3).

We found that 1 and 2 h were needed to consume the extra 500 μg/mL arginine for *L. brevis* and *W. confusa*, respectively. *L. sakei* cultures showed a higher citrulline content than the MRS medium at all time points. *P. pentosaceus* showed increased citrulline contents that paralleled bacterial growth. *L. plantarum*, *L. lactis,* and *L. mesenteroides* did not show increased citrulline production after the addition of arginine (Figure 3). However, the ornithine content was higher in the culture medium with extra arginine in all lactic acid bacteria cultures. The citrulline and ornithine contents were consistent with arginine consumption in the *L. brevis* and *W. confusa* cultures. The arginine content dramatically decreased in both lactic acid bacteria cultures. Arginine was exhausted after 3 or 4 h of culture with *L. brevis* (in MRS medium without or with added arginine, respectively). *W. confusa* showed relatively slower arginine catabolism than *L. brevis*. The arginine supply was exhausted after 5 or 7 h in the *W. confusa* culture (Figure 3). Citrulline and ornithine production by *L. brevis* and *W. confusa* showed a pattern that was consistent with arginine catabolism. The citrulline concentration of *L. brevis* increased to a maximum at around 1–3 h after growth was initiated, and the maximum concentration of 98.8 ± 1.6 μg/mL was observed at 2 h after the addition of arginine. *W. confusa* showed a difference in citrulline production when compared to *L. brevis.* Interestingly, *P. pentosaceus*, *L. plantarum*, *L. mesenteroides*, and *L. lactis* did not show increased citrulline after arginine was added. The ornithine contents were higher in all bacteria except for *L. lactis* after the addition of arginine to the culture (Figure 3). Adding arginine increased the pH slightly at the starting point of cultivation, but did not affect the pH during growth. The pH changes in cultures of *L. brevis*, which has strong arginine-catabolism activity, were slower than those in the control or in other lactic acid bacteria (Figure 3), suggesting that *L. brevis* produced more ammonia.

### 2.4. Effect of Citrulline and Ornithine on the Growth of Lactic Acid Bacteria

**Figure 4:**
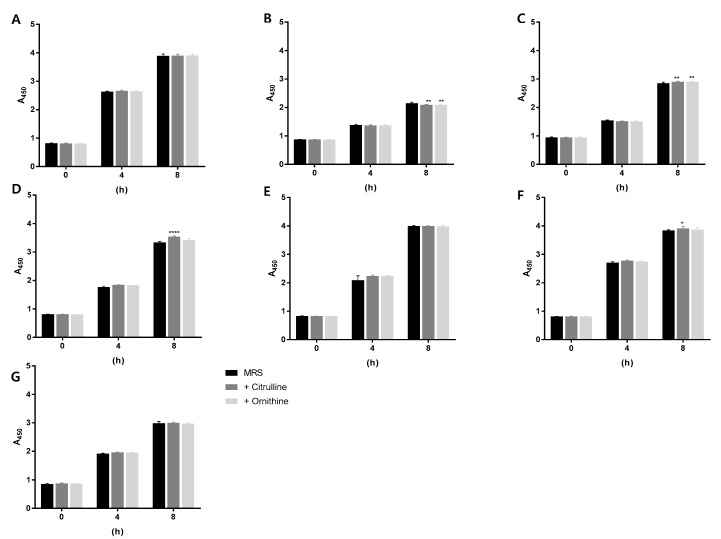
Effects of added arginine, citrulline, and ornithine on the growth of lactic acid bacteria. Bacterial growth with the addition of 1.5 mM citrulline and ornithine was monitored by performing WST-8 colorimetric assays. After a 4 or 8 h incubation at 30 °C, the absorbance of the samples in the presence of the WST-8 was measured at 450 nm. (**A**) *L. brevis*; (**B**) *P. pentosaceus*; (**C**) *L. plantarum*; (**D**) *L. lactis*; (**E**) *L. sakei*; (**F**) *L. mesenteroides*; (**G**) *W. confusa*. Here, * *p* < 0.05, ** *p* < 0.01, **** *p* < 0.0001.

Bacterial growth was monitored using the WST-8 method [13,14] to investigate the effect of arginine on bacterial growth. MRS media were prepared with an equivalent amount of arginine in the MRS medium by adding 1.5 mM of citrulline or ornithine. The bacteria were cultivated at 30 °C, and the growth was monitored every 4 h. Interestingly, the addition of citrulline increased the growth of *L. lactis* and *L. mesenteroides* more than growth in an MRS (1.5 mM arginine) medium or ornithine (Figure 4).

### 2.5. Arginine Catabolism in Kimchi

**Figure 5:**
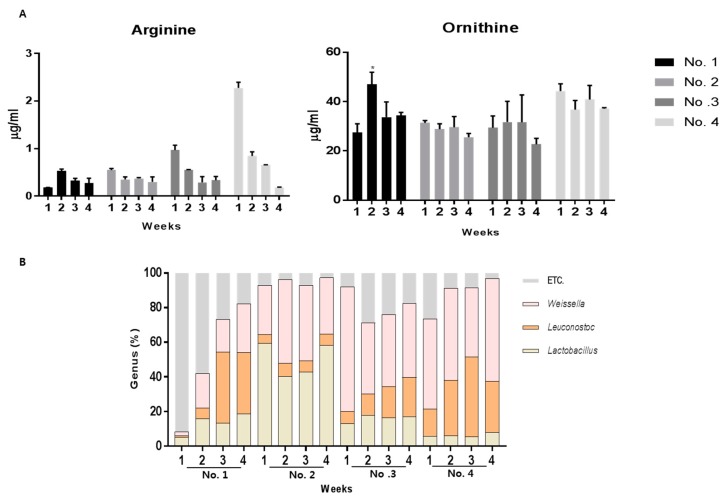
Arginine catabolism during kimchi fermentation. (**A**) Quantification of arginine and ornithine production in kimchi; (**B**) comparison of bacterial communities among the commercial kimchi samples. All kimchi samples were purchased from a local market.

The commercial kimchi samples were analyzed to monitor citrulline and ornithine production. Kimchi samples no. 3 and no. 4 showed a decrease in arginine concentrations to 0.3 ± 0.1 μg/mL and 50.2 ± 0.1 μg/mL, respectively. The ornithine concentration ranged from 22.7 ± 10 μg/mL to 44 ± 2 μg/mL in the 1-week kimchi samples (Figure 5A). Metagenomics data analysis showed the distribution of lactic acid bacteria in kimchi (Figure 5B). Kimchi sample no. 1 (at 1 week) showed a small proportion of lactic acid bacteria in the kimchi, and the ornithine concentration was dramatically increased by incremental increases in the lactic acid bacteria abundance in the kimchi samples.

## 3. Discussion

Kimchi is a fermented vegetable food, and lactic acid bacteria serve as important drivers of kimchi fermentation by converting raw materials into diverse metabolites (such as organic acids and sugars) and active compounds (such as mannitol, which is an important factor in kimchi taste and is converted from fructose by heterofermentative lactic acid bacteria).

The physiological properties of kimchi are well characterized as a representative fermented Korean food. Kimchi ingredients also act as a source of nutrition for bacteria during fermentation. Carbohydrates represent an important source of raw materials for producing diverse organic acids, including lactic acid. Aas are also important for the growth of bacteria. Carbohydrates and aas are the primary nutritional resources required for bacterial growth.

Kimchi contains various compounds originating from raw materials, such as exopolysaccharides, vitamins, phenolic compounds, γ-amino butyric acid, and mannitol, as well as organic acids, which have been reported to originate from lactic acid bacteria [1]. Recently, we identified leucine as a metabolite in kimchi. Leucine is catabolized in kimchi by dominant lactic acid bacteria such as *Lactobacillaceae* and *Leuconostocaceae*. The content of the leucine derivative, 2-hydroxyisocaproic acid, was positively correlated with lactic acid bacteria content during the early stage of kimchi fermentation [5]. Interestingly, the leucine content did not change significantly during kimchi fermentation, indicating that leucine was sufficient to produce HICA during fermentation [5]. The aa content was characterized during kimchi fermentation [11,12,19,20]. The aa content in kimchi varied during fermentation, although the aa changes were not significant during fermentation. The addition of fish sauce, which is generally regarded as an important protein source for kimchi, did not increase the aa content in kimchi significantly [11].

In contrast to the content of other aas in kimchi, the arginine content in kimchi decreased significantly during fermentation [10,20] or was nearly exhausted over the fermentation period [12].

Arginine is an essential aa in mammals and bacteria. Arginine is important for several medical and industrial applications; stimulates the secretion of growth hormones, prolactin, and insulin; and helps maintain muscle mass. Arginine is also an important component of the urea cycle and is metabolized into citrulline and ornithine through the ADI pathway in bacteria. Arginine catabolism during alcohol fermentation has been well characterized. Wine fermentation has showed a close relationship with arginine content, bacterial growth, and acid resistance [21], especially malolactic acid fermentation in grape must and wine [14,15].

Although nutrition and adaptation are important for bacterial growth, arginine catabolism by lactic acid bacteria during kimchi fermentation has not been characterized yet. In this study, we analyzed the arginine-catabolism activity of lactic acid bacteria and evaluated citrulline and ornithine production associated with the ADI pathway and the effects on bacterial growth.

To characterize the arginine-catabolism acidity, we investigated aa catabolism by lactic acid bacteria originating from kimchi using LC-MS. Interestingly, two lactic acid bacteria, *L. brevis* and *W. confusa*, showed significantly decreased arginine levels in the culture media.

Lactic acid bacteria also have an ADI pathway that is involved in producing citrulline and ornithine from arginine. Arginine is an essential aa in humans, rodents, and prokaryotes. Arginine is synthesized from citrulline in prokaryotes and eukaryotes. Aas also serve as an energy source for bacteria. For example, many lactic acid bacteria use arginine and citrulline as an energy source via the ADI system [22].

The ADI pathway is widely utilized in bacteria. The composition of ADI pathway enzymes and transporters varies among different bacterial groups [23]. Among 72 *Lactobacillales* members studied, 32 had ADI enzymes [16]. The ADI pathway comprises arginine deiminase (ADI, *arcA*), ornithine transcarboxylase (OTC, *arcB*), and carbamate kinase (CK, *arcC*). ADI converts l-arginine into l-ornithine and yields 1 mol of ATP per mol of arginine [22]. ADI converts arginine into citrulline and ammonia. Subsequently, the carbamoyl moiety of citrulline is transferred to OTC and yields ornithine and carbamoyl phosphate. CK yields carbon dioxide, ammonia, and ATP [23]. The arginine to ornithine antiport (*arcD*) system is essential for recycling arginine and citrulline during energy generation [22].

Among the seven lactic acid bacteria used in this study, six possess the *arcA* gene. *L. lactis* does not have *arcA*, but does have *arcB* and *arcC*. *L. plantarum* ADI_LP_ shares a similarity with *W. confusa* ADI_WC_, although the aa sequence of ADI_LP_ (229 aa) is shorter than that of other ADI proteins (*W. confusa*: 412 aa; *P. pentosaceus*: 407 aa; *L. brevis*: 410 aa; *L. mesenteroides*: 319 aa; *L. sakei*: 411 aa) (Figure 1A). The seven lactic acid bacteria studied here have OTC and high ornithine production activities, except for *L. lactis* (Figure 3).

Arginine catabolism-related genes include *adi*, which is required for disassociation of arginine into citrulline and ammonia. The arginine- and citrulline-catabolism pathways of *L. brevis* have been reported recently [22]. The ADI pathway comprises a cluster of three genes (*arcD1*, *arcE1*, and *arcE2*) [22]. Arginine facilitates the growth of *L. brevis*, especially during wine fermentation. *L. brevis* uses arginine and citrulline as energy, carbon, and nitrogen sources, as well as for survival in acidic environments. Supplementation with arginine or citrulline has shown an enhancement of bacterial growth [22]. Especially, *L. lactis* and *L. mesenteroides* growth increased after adding citrulline (Figure 4). These findings suggest that citrulline affected the growth of ADI-deficient incomplete lactic acid bacteria, which could not use arginine. Table 2 shows the distributions of ADI and OTC among the lactic acid bacteria, as well as evidence suggesting that citrulline was important for the growth of lactic acid bacteria. Data generated in this study clearly suggest that arginine is an essential aa for the fermentation of foods such as kimchi. Arginine is rich in garlic, known to be an important source of lactic acid bacteria in kimchi [6,24], suggesting that arginine-rich ingredients such as garlic play important roles as energy sources during kimchi fermentation and further influence bacterial ontology in kimchi fermentation environments.

To compare the lactic acid bacteria population and arginine catabolism in kimchi, we quantified the arginine, citrulline, and ornithine contents in commercial kimchi samples obtained from the market (Figure 5). The overall arginine concentration was lower than the reported concentration. Four kimchi samples had arginine concentrations ranging from 0.2 μg/mL to 2 μg/mL. Previous reports showed that the arginine content in kimchi was 50–60 μg/mL and decreased to 1–2 μg/mL over the course of fermentation [12]. The small amount of arginine in the kimchi detected in this study may reflect arginine consumption, a possibility supported by observing higher ornithine contents. Citrulline was not detected in the four kimchi samples. As shown in Figure 3, citrulline was rapidly converted into ornithine by lactic acid bacteria. The ornithine content showed consistent results with metagenomics analysis. Especially, the ornithine content significantly increased (*p* < 0.05) in kimchi sample no. 1 after two weeks, and the lactic acid bacteria composition also increased (Figure 5B). However, the ornithine content in the other kimchi samples did not increase significantly, and the lactic acid bacteria composition already represented 70%–80% of the total bacteria.

The ADI system produced ammonia, an important source of nutrients for bacteria as a nitrogen source that is used to make proteins and nucleic acids and that serves as an important source of nutrients for bacteria during aa and nucleic acid synthesis. Recent studies on ammonia have indicated that ammonia is related to bacterial responses and is critical for bacterial sensing [17,18].

Metabolite production (or bioconversion) is important to bacterial adaptation to changes in nutrition and to contributing to the flavor of fermented foods. In a previous study, the ornithine contents in kimchi increased when arginine and citrulline contents decreased [11]. These results also support our finding that arginine catabolism was important for kimchi fermentation and that citrulline may have acted as an energy source in ADI-deficient bacteria during kimchi fermentation.

In this study, we found that ornithine production in kimchi was related to arginine consumption by lactic acid bacteria, suggesting that the growth of lactic acid bacteria in the kimchi was related to arginine catabolism and that arginine and citrulline served as an energy source associated with ADI or OTC activity.

## 4. Materials and Methods

### 4.1. Medium and Bacterial Culture Conditions

*L. brevis, L. mesenteroides, P. pentosaceus, L. lactis, W. confusa, L. plantarum*, and *L. sakei* were previously identified in kimchi by 16S rDNA sequencing [5,25]. The bacteria were cultivated in MRS broth. To compare arginine-dependent citrulline and ornithine production, MRS was prepared with an additional 500 μg/mL of arginine. To monitor the effects of arginine metabolites such as citrulline and ornithine, MRS was prepared with additional citrulline and ornithine (1.5 mM each). Bacterial growth was monitored spectrophotometrically by measuring the absorbance at 600 nm, and colorimetric assays were performed using the WST-8 Cell Proliferation Assay Kit at 450 nm after a 4 or 8 h incubation at 30 °C, as described previously [25].

### 4.2. Sample Preparation and Analysis

The arginine, citrulline, and ornithine contents were measured in cell-free culture supernatants and in kimchi. Each bacterial culture was harvested by centrifugation at 12,000 rpm for 10 min, and cell-free clear supernatant was extracted using 40% acetonitrile in the presence of an internal standard (l-leucine-5,5,5-d3, final concentration of 0.5 mM) and centrifuged to remove the insoluble fraction. Kimchi samples were prepared by homogenization, and insoluble fractions were removed by centrifugation at 12,000 rpm for 10 min. Clear fractions were extracted in the same manner as described for the bacterial samples. Standard solutions of arginine, citrulline, and ornithine were individually prepared at concentrations of 100, 250, 500, 750, and 1000 μM.

A TripleTOF 5600 plus instrument (SCIEX, Redwood City, CA, USA) coupled with an Acquity UPLC system (Waters, Milford, DE, USA) equipped with an ACQUITY BEH Amide column (2.1 × 100 mm, 1.7 μm) was used to characterize the metabolites and quantify the contents of bacteria. Mobile phase A (10% acetonitrile with 10 mM ammonium acetate) and mobile phase B (90% acetonitrile with 10 mM ammonium acetate, pH 9) were used. The ultraperformance liquid chromatography gradient program started with 0.1% A and 99.9% B at a flow rate of 0.4 mL/min. The analysis was performed in negative ionization mode. The collision energy was –30 eV. The source temperature was 300 °C. The injection volume was 1 µL. The MRM conditions generated the following transitions: Arginine, *m*/*z* 173 > 131; citrulline, *m*/*z* 174 > 131; ornithine, *m*/*z* 131 > 131; l-leucine-5,5,5-d3 (as the internal standard), *m*/*z* 133 > 133. For the internal standard, l-leucine-5,5,5-d3 (final concentration 0.5 mM) was spiked into the sample. The peak areas for the acquired data were normalized with the internal standard and processed using MultiQuant (SCIEX).

### 4.3. Protein Sequence Analysis

The ADI and OTC aa sequences were collected from the UniProt Protein Sequence Database. The sequences were aligned using the clustalW server (https://embnet.vital-it.ch/software/ClustalW.html) blosum matrix. The alignment results were shaded to show similarities with GenDoc software, and similarity and phylogenic trees were generated using the CLC genomics workbench program, version 7.5.

### 4.4. Statistical Analysis

All experiments were performed in triplicate. The data are presented as means and standard derivations. A two-way analysis of variance test was performed using GraphPad Prism software with Tukey’s multiple-comparison test.

### 4.5. Comparison and Analysis of Metagenomics Data

Metagenomics analysis was performed using DNA isolated from commercial kimchi. The samples were analyzed by ChunLab, Inc. (Seoul, Korea), using the Illumina MiSeq sequencing system (Illumina, San Diego, CA, USA) in accordance with the manufacturer’s instructions. The operational taxonomic units among the samples were compared and analyzed using the CL community program (ChunLab Inc., Seoul, Republic of Korea).

## Figures and Tables

**Table 1:** Characterization of the ADI system in lactic acid bacteria.

Bacterial Strain	ADI		OTC		CK	
	Protein	Gene	Protein	Gene	Protein	Gene
*L. brevis* ATCC 367/JCM 1170	Q03NY8	*arcA*, (LVIS_20270	Q03NY9	*arcB*, (LVIS_2026)	Q03NG5, Q03NZ2	LVIS_2207, LVIS_2023
*P. pentosaceus* ATCC 25754	Q03DS2	*arcA*, (PEPE_1629)	Q03DS0	*arcB*, (PEPE_1631)	Q03DS1, Q03HM6	PEPE_1630, PEPE_0192
*L. plantarum* WCFS1	Q6U3A0	*arcA*	O08322	*argF*, (lp_0532)	Q6U398	*arcC*
*L. lactis*	-	-	A0A1B2A277	BCR17_06740	-	-
*L. sakei* 23K	Q38YQ6	*arcA*, (LCA_0370)	Q38YQ5	*arcB*, LCA_0371	Q38YQ4	*arcC*, (LCA_0372)
*L. mesenteroides* ATCC 8293	Q03XZ3	*arcA*, (LEUM_0821)	Q03W72	*arcB*, (LEUM_1457)	-	-
*W. confuse* LBAE C39-2	H1X7H9	*arcA*, (WEISS39_05260)	H1X7H8	*arcB*, (WEISS39_05255)	H1X7H6	*arcC3*, (WEISSC39_05245)

Characterization of ADI system proteins and their corresponding genes in lactic acid bacteria. ADI: arginine deiminase; OTC: ornithine transcarbamylase; CK: carbamate kinase.

**Table 2:** Summary of the ADI systems present in various lactic acid bacteria.

Bacterial Strain	ADI	OTC	CK
*Lactobacillus brevis*	●	●	●
*Lactobacillus curvatus*			
*Lactobacillus graminis*			
*Lactobacillus mali*		●	
*Lactobacillus parabrevis*		●	●
*Lactobacillus parabuchneri*	●	●	●
*Lactobacillus paracasei*			
*Lactobacillus paraplantarum*		●	
*Lactobacillus pentosus*		●	
*Lactobacillus plantarum*	●	●	●
*Lactobacillus sakei*	●	●	●
*Lactococcus lactis*	●	●	●
*Leuconostoc carnosum*			
*Leuconostoc citreum*		●	
*Leuconostoc gelidum subsp. gasicomitatum*		●	
*Leuconostoc kimchii*		●	
*Leuconostoc lactis*		●	
*Leuconostoc mesenteroides*	●	●	
*Leuconostoc pseudomesenteroides*		●	
*Pediococcus pentosaceus*	●	●	●
*Streptococcus gallolyticus*	●	●	
*Weissella cibaria*	●	●	●
*Weissella confusa*	●	●	●
*Weissella hellenica*			
*Weissella paramesenteroides*			
*Weissella soli*			
*Weissella viridescens*	●		
*Weissella koreensis*	●	●	●

Comparison of the ADI system in lactic acid bacteria. ADI: arginine deiminase; OTC: ornithine transcarbamylase; CK: carbamate kinase. The proteins were identified using the UniProt Protein Database.

## Abbreviations

aa	Amino acid
ADI	Arginine deiminase
CK	Carbamate kinase
HICA	2-Hydroxyisocaproic acid
MRM	Multiple-reaction monitoring
MRS	De Man, Rogosa, and Sharpe
OTC	Ornithine transcarbamylase
